# BGFE: A Deep Learning Model for ncRNA-Protein Interaction Predictions Based on Improved Sequence Information

**DOI:** 10.3390/ijms20040978

**Published:** 2019-02-23

**Authors:** Zhao-Hui Zhan, Li-Na Jia, Yong Zhou, Li-Ping Li, Hai-Cheng Yi

**Affiliations:** 1 China University of Mining and Technology, Xuzhou 221116, China; TS16170022A3@cumt.edu.cn (Z.-H.Z.); yzhou@cumt.edu.cn (Y.Z.); 2 College of Information Science and Engineering, Zaozhuang University, Zaozhuang 277100, Shandong, China; 3 Xinjiang Technical Institute of Physics and Chemistry, Chinese Academy of Sciences, Urumqi 830011, China; Lipingli@ms.xjb.ac.cn (L.-P.L.); yihaicheng17@mails.ucas.ac.cn (H.-C.Y.)

**Keywords:** ncRNA-protein interaction, bi-gram, position specific scoring matrix, k-mers, deep learning

## Abstract

The interactions between ncRNAs and proteins are critical for regulating various cellular processes in organisms, such as gene expression regulations. However, due to limitations, including financial and material consumptions in recent experimental methods for predicting ncRNA and protein interactions, it is essential to propose an innovative and practical approach with convincing performance of prediction accuracy. In this study, based on the protein sequences from a biological perspective, we put forward an effective deep learning method, named BGFE, to predict ncRNA and protein interactions. Protein sequences are represented by bi-gram probability feature extraction method from Position Specific Scoring Matrix (PSSM), and for ncRNA sequences, k-mers sparse matrices are employed to represent them. Furthermore, to extract hidden high-level feature information, a stacked auto-encoder network is employed with the stacked ensemble integration strategy. We evaluate the performance of the proposed method by using three datasets and a five-fold cross-validation after classifying the features through the random forest classifier. The experimental results clearly demonstrate the effectiveness and the prediction accuracy of our approach. In general, the proposed method is helpful for ncRNA and protein interacting predictions and it provides some serviceable guidance in future biological research.

## 1. Introduction

In recent studies, non-coding RNA (ncRNA) plays a regulatory role in controlling cell molecules, which gradually attracts researchers’ attentions. In the field of known biological knowledge, ncRNAs are interpreted as those RNAs that are transcribed from the genome but cannot be translated into proteins. Therefore, the functions of these ncRNAs are vastly different. In other words, each ncRNA has its own role in the processes of protein translations, which is extremely confusing for most researchers. In recent works, more and more evidences indicate that the occurrences of a series of major diseases are related to the disequilibrium of ncRNAs. Meanwhile, increasing amounts of ncRNAs whose functions have not been known yet are discovered through some advanced technologies [1,2,3]. Therefore, it is urgent to make the biological functions of these ncRNAs, such as RNA stability and RNA translation, clear. To learn about the functions of the ncRNA, researchers need to confirm whether ncRNAs are able to interact with other proteins in the processes of biological reactions [4]. Shen et al. proposed a method to predict ncRNA and protein interactions based on sequences by way of deep learning named IPMiner (Interaction Pattern Miner) [5]. Furthermore, several machine learning techniques, including support vector machine (SVM), have been put forward in predicting RNA-binding residues in proteins [6].

Recently, great progresses have been made in the study of RNA-protein interactions [2,7,8,9,10,11,12,13,14]. Although excellent achievements have been obtained both in the field of supervised and unsupervised learning, there still are some shortcomings and spaces for improvement in the current methods. The high-throughput technologies consume too much time, the determination of RNA’s complex structure requires a large amount of physical resources [12], and some sequence specificity methods fail to predict the exact interaction of ncRNAs and proteins. Since the sequence specificity of ncRNA and protein interactions have been shown by a large number of studies, it is indicated that the sequence has been able to carry enough useful information to predict the interaction between ncRNA and proteins [7,15]. Therefore, extracting feature information from sequences is considered to be a reliable and effective method that can discern whether the ncRNA and protein are capable of reacting each other well. In order to obtain more accurate prediction results, some innovative techniques that are only based on the sequences of ncRNA and proteins were proposed in predicting RNA-protein interactions. Suresh V et al. reported the interactions between ncRNA and proteins can be well predicted according to cumulative experimental validation [14]. In addition, some other studies focus on the interface of ncRNA and protein in proteins that can indicate how the reactions interact each other [16]. Yi et al. also proposed a deep learning framework, named RPI-SAN, using pure sequence information and employed complex stacked auto-encoder network in predicting these interactions [17].

In this study, we put forward a sequence-based method using deep learning model Stacked auto-encoder (SAE) network combined with Random Forest (RF) classifier. We used k-mers sparse matrices to represent ncRNA sequences and then extracted feature vectors from these matrices by Singular Value Decomposition (SVD). For protein sequences, to excavate more biological information, Position Specific Scoring Matrix (PSSM) was used to obtain evolutionary information from each sequence; moreover, a bi-gram algorithm was further used to get feature vectors from PSSMs. As the advantage of deep learning is representation learning, which means that learning representations of data make it easier to extract useful information when building classifiers or other predictors. SAE was further employed to learn high-level hidden information. Subsequently, data and labels were all fed into the RF classifier to classify whether a pair of protein and ncRNA interacted or not. Furthermore, to evaluate the performance of our approach, five-fold cross-validation and generic evaluation measures were used. We also compared our method BGFE with other methods on three benchmark datasets. According to the experimental results, BGFE performed much better than other methods, with the specific accuracies of 0.8868, 0.9600, and 0.9130 on dataset RPI488, RPI1807, and RPI2241, respectively. The experimental results show that our method achieved high accuracy and robustness of the protein-ncRNA interaction prediction task.

## 2. Results

**Figure 1:**
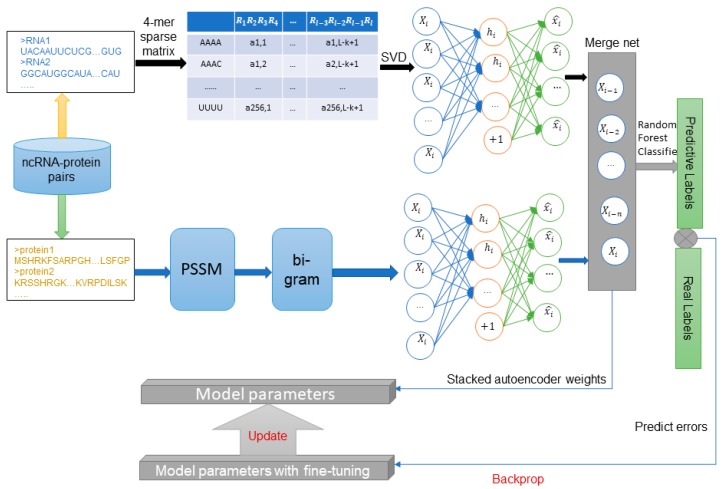
Step-wise work flow for the proposed BGFE method. In the non-coding RNA (ncRNA) and protein sequences used for training and prediction, Singular Value Decomposition (SVD) converts ncRNA sequences into feature vectors from 4-mer sparse matrices, while protein sequences are represented by bi-gram algorithm form Position Specific Scoring Matrix (PSSM). These feature vectors are processed by multi-layer stack auto-encoder to obtain deeper feature information. Subsequently, training data and labels are fed into a random forest classifier for classification training. In addition, fine-tuning the model parameters after obtaining the machine learning model further contributes the model accuracy.

In this study, we purposed a sequence-based method using deep learning model SAE network combined with the RF classifier that was named BGFE. Figure 1 shows the workflow for this method.

### 2.1. Performance Evaluation

As shown in Table 1, the specific performance is composed of five indicators, including accuracy, sensitivity, specificity, precision, and MCC, respectively [18]. Our method BGFE achieved a performance with the accuracy of 0.8868, sensitivity of 0.9268, specificity of 0.8354, precision of 0.9328, and MCC of 0.7744 on dataset RPI488. On dataset RPI1807, the result reached at a high accuracy of 0.9600, sensitivity of 0.9344, specificity of 0.9989, precision of 0.9117, and MCC of 0.9217. On dataset RPI2241, the predicting result was up to the accuracy of 0.9130, sensitivity of 0.8772, specificity of 0.9660, precision of 0.8590, and MCC of 0.8335.

**Figure 2:**
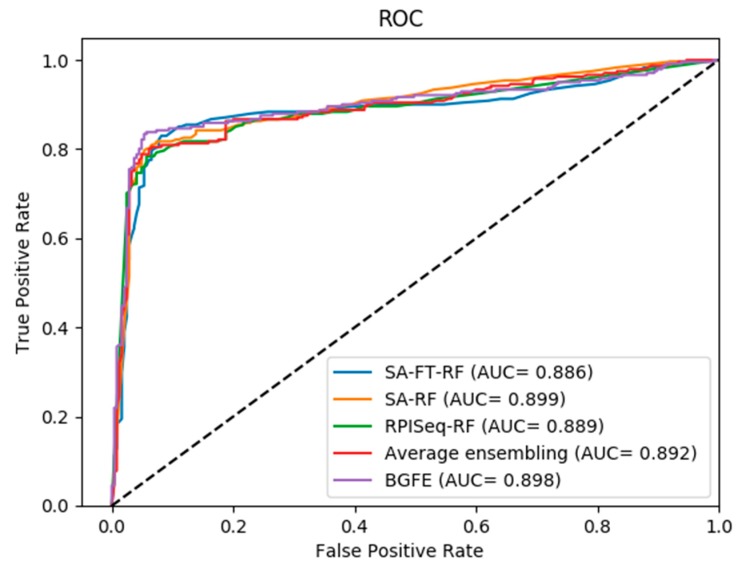
ROC curves of performance comparisons between BGFE and other strategies on dataset RPI488.

**Figure 3:**
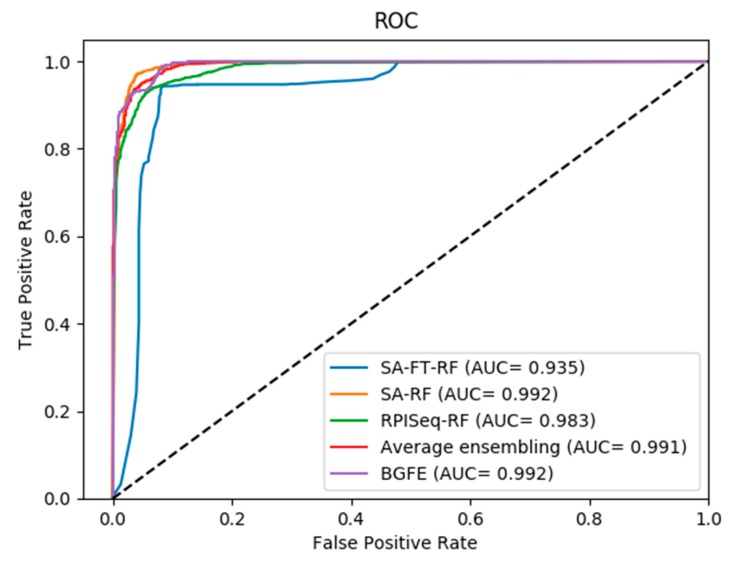
ROC curves of performance comparisons between BGFE and other strategies on dataset RPI1807.

**Figure 4:**
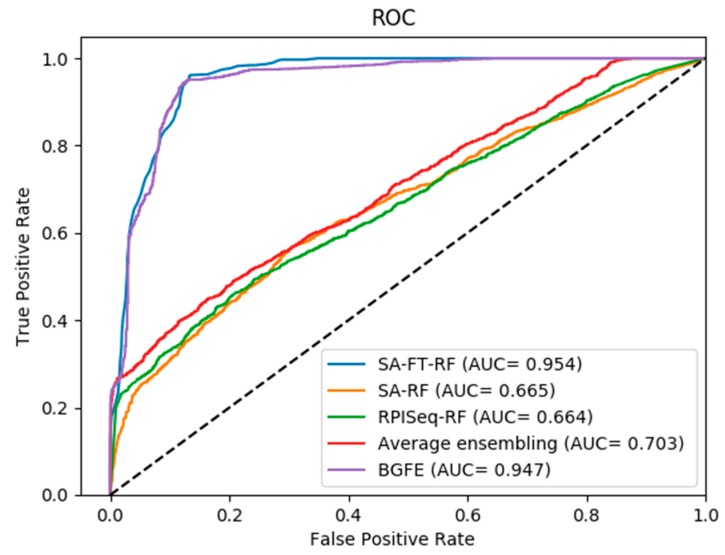
ROC curves of performance comparisons between BGFE and other strategies on dataset RPI2241.

On the other hand, as shown in the Receiver Operating Characteristic (ROC) curves in Figure 2, Figure 3 and Figure 4, the corresponding AUC of RPI488, RPI1807, and RPI2241 are 0.8980, 0.9920, and 0.9470, respectively. From the experimental results, we can figure out that the accuracy has been increased to ninety percent on dataset RPI2241, which shows that high level protein features can be directly extracted from PSSM instead of counting the frequency of occurrence of amino acids from the protein consensus sequences and primary sequences.

### 2.2. Comparison between Three Base Models and Final Integration Model BGFE

In this study, we used stacked ensembling to integrate three base predictors as the final predictor. In order to prove the superiority of this ensembling strategy, we compared BGFE with three base models, named SA-RF, SA-FT-RF, and RPIseq-RF. The results are reported in Table 2, Table 3 and Table 4. In particular, SA-FT-RF means the stacked autoencoder with Fine Tuning and Random Forest classifier. Similarly, SA-RF means Stacked autoencoder with Random Forest classifier (without fine tuning), and RPIseq-RF means the Random Forest version RPIseq using raw feature and these three models are base predictors for the stacked ensambling strategy. Three basic models are integrated as the final model, named BGFE.

As shown in Figure 2, Figure 3 and Figure 4, the ROC curves indicate the intuitive comparison among SA-FT-RF, SA-RF, RPIseq-RF, and the proposed BGFE. The *x*-axis of the ROC curve represents the false positive rate, while the *y*-axis of the ROC curve expresses the true positive rate. According to the results of the comparison, we found that our ensembling strategy had a better prediction accuracy when compared to other three base models at the specific accuracies of 0.8868 and 0.9130 in RPI488 and RPI2241, respectively. In RPI1807 dataset, although BGFE did not achieve the best performance, it still has an accuracy of 0.9600. This comparison results revealed that our method with stacked ensembling had a good performance in predicting interactions from base models, especially for those datasets with lower correlation.

### 2.3. Comparison of Prediction with BGFE and Other Methods

To further verify the reliability of our method BGFE, we also compared BGFE with other experimental methods at the present stage. V. Suresh et al. proposed a computational method to predict ncRNA and protein interaction, named RPI-Pred, by using the sequence and structural information [14]. RPI-Pred adopted the well-known SVM classifier, which was implemented as an independent in-house procedure. On this basis, RPI-Pred was evaluated by using a 10-fold cross-validation (10-fold CV) rather than the five-fold cross-validation in BGFE. The accuracy of these two cross validation methods is similar, but, in general, five-fold CV is used more widely in biological experiments. Usha K Muppirala et al. also proposed a sequence-based method similarly to our method, but adopted different feature extraction methods [19]. They used two classifiers including SVM and RF to fit the training dataset and predict the interactions between ncRNAs and proteins. Here, we only compared their performance by using an RF classifier instead of SVM. Ying Wang et al. proposed a novel extended naive-Bayes-classifier to predict ncRNA and protein interactions only based on sequence as well [20]. The classifier that they used was quite different from the traditional one. They extracted effective features by reducing the likelihood ratio score, which can not only integrate the transparent features, but also reduce the order of computational complexity in the process of predictions. Table 5 shows the comparison between BGFE and other three methods.

When compared with these four methods, our method BGFE resulted in a better performance both on dataset RPI1807 and the RPI2241. BGFE achieved an AUC of 0.9970 in RPI1807 and 0.9640 on RPI2241, respectively. The high precision prediction results indicated that only extracting features from sequence was reliable and credible in predicting ncRNA and protein interactions.

## 3. Discussion

In this study, we presented a computational scheme to mine the deep ncRNA-protein interaction patterns and then predict them based on stacked auto-encoder and stacked ensembling. It has achieved accurate and reliable accuracies of 0.8868 on dataset RPI488, 0.9600 on dataset RPI1807, and 0.9130 on dataset RPI2241, respectively. These comprehensive experimental performances on datasets with different characteristics proved the effectiveness of BGFE well. The comparison results between BGFE and other basic models also indicate that discriminant high-level features automatically learned from multiple layers of neural network can be well extracted by auto-encoder.

The proposed method BGFE resulted in the reliable ncRNA and protein interaction prediction performance with high accuracies, which have mainly benefited from the following points in our opinion: (1) The use of PSSM ensures that the effective and useful information can be extracted from the protein sequences and makes these feature-information simple and intuitive. (2) From a biological point of view, the bi-gram feature extraction method can help us to distinguish those protein folds from the different amino acids subsequences in the conserved areas. As a result, there is an individual group of bi-gram features that represents each protein sequence in the conserved area. (3) Deep learning is a tool to model complicate statistical features in datasets. Therefore, through deep learning, hidden relationships between k-mers sequence motif can be well automatically learned by a stacked auto-encoder. The specific mechanism of stacked auto-encoder accurately identifies and extracts the most informative hidden-level features, and meanwhile eliminating the hidden irrelevant variabilities to avoid cures of dimensionality. The high-dimensional raw protein and ncRNA features especially demand this kind of dimensionality reduction and feature simplification. (4) On the other hand, stacked ensembling is able to integrate individual strengths of different predictors well, which provides better performances than previous manually designed average voting and majority voting. 

However, actually, our approach is currently obtained by training small-scale datasets, and only a minor part of ncRNA and protein interaction patterns in nature are verified in this experiment, because of the difficulty in collecting large-scale complex interactions from nature and corresponding databases. On the other hand, as we all know that the bigger dataset gives the better performance of deep learning because of the automatic learning of sequences’ representative features. Accordingly, training datasets as big as possible are inquired to take all the possible situations into consideration. In order to achieve this goal, a large number of the datasets that were established by positive samples ought to be collected from the structure complexes or from other experimental methods. At the same time, the negative samples will also verify their importance in predicting performance. Hence, we should also figure out an efficient method in learning negative samples distributions of ncRNA and protein pairs. In addition, another strategy to cope with sample unbalance is to train the model with similarity matrices only using positive samples.

In general, although our method BGFE performs well in predicting interactions between ncRNA and proteins, like other deep learning algorithms, there are a lot of limitations and disadvantages. There is still much room for improvement in our research. Predicting interaction methods is a black box about learning machines without the biological in-sight in the ncRNA and protein pairs. Our method tries to extract protein features and automatically learn the advanced features with the help of random forests classifier, but it still does not make a very good breakthrough from the perspective of biology. In the future research, we will expect the design of a better network architecture for extracting hidden advanced features from the perspective of biology.

## 4. Materials and Methods

### 4.1. Datasets

We executed experiments on three public datasets, including RPI488, RPI1807, and RPI2241. The dataset RPI488 was purposed in IPMiner [5,21]. It is a non-redundant long ncRNA-protein interaction dataset that is based on structure complexes, which consists of 488 protein-lncRNA pairs, including 243 interactive pairs and 245 non-interactive pairs. On the other hand, two more sets of data, RPI1807 and RPI2241, were directly collected from the RPIseq database [12,14]. The RPI1807 is established by parsing the Nucleic Acid Database (NAD), which contains 1807 positive ncRNA-protein interaction pairs, which includes 1078 RNA chains, 1807 protein chains, and 1436 negative ncRNA-protein interaction pairs, which includes 493 RNA chains and 1436 protein chains, respectively. While, RPI2241 consists of 2241 ncRNA and protein pairs. For these three datasets, they were all extracted from structure-based complexes. The datasets details show as following Table 6.

### 4.2. Position Specific Scoring Matrix

Position Specific Scoring Matrix (PSSM) is capable of testing the distantly related proteins in past studies. From the biological perspective, PSSM is a matrix that is used to distinguish the similarity of two sequences, since PSSM are able to predict quaternary structural attributes, protein disulfide connectivity, and folding pattern [22,23,24]. Each element of the PSSM indicates the probability of the substitution of an amino acid to another amino acid [25]. If the replacement of these two amino acids is frequent, then it indicates that this substitution can be accepted by nature with high amino acid substitution scores [26]. Each random protein sequence can be transformed into a PSSM through the Position Specific Iterated BLAST (PSI-BLAST) [27].

Let *P* be a PSSM as the representative of an arbitrary protein. BLAST software that is characterized by executing the parameter command about related proteins executes the PSSMs. A PSSM consists of *r* rows and 20 columns with the explanation that *r* means the length of the primary sequence of an arbitrary protein, while 20 means the quantity of amino acids, respectively.

(1) $$P=\left\{p_{i,j}\;i=1\ldots r,j=1\ldots 20\right\}$$

The element in a PSSM at the position of *i* row and *j* column is represented by the symbol $P_{i,j}$. The symbol $P_{i,j}$ ($\sum_{j=1}^{20}p_{i,j}=1,for\;i=1,2,\ldots ,r$) denotes the relative probability of $j_{th}$ amino acid at the $i_{th}$ position of the same protein sequence with which PSSM comes from [28,29].

In this study, PSI-BLAST software was used to transform each random protein sequence into a PSSM the same as most studies for creating the train datasets and predicting interactions. In order to obtain the protein sequences with higher and broader homology, the parameter *e-value* of PSI_BLAST method was set to 0.001 [30]. Subsequently, three iterations are used to obtain the completed PSSMs from protein sequences.

### 4.3. Bi-gram Feature Extraction of PSSM

To extract the features recognized from the protein folds, a bi-gram feature extraction technique is employed by way of PSSM linear probabilities. In the meantime, we are incapable of directly extracting bi-gram features from the protein represented by the primary sequences or the consensus sequences, because the combinations of amino acid cannot all be found in the protein sequences [31]. Therefore, PSSM is figured out to replace it. Meanwhile, the bi-gram feature vector is computed through the representing information that is mainly contained from PSSM [32]. A more specific mathematical explanation will be given in the following paragraphs.

Let *B* be a bi-gram occurrence matrix and $b_{m,n}$ be the element in the matrix *B*. The symbol $b_{m,n}$ can be interpreted as the occurrence probability of the transition from $m_{th}$ amino acid to $n_{th}$ amino acid that is able to be calculated from the element $P_{i,j}$ in its PSSM as the following equation:

(2) $$B=\left\{b_{m,n},1\leq m\leq 20,1\leq n\leq 20\right\}$$

(3) $$b_{m,n}=\sum_{i=1}^{r-1}p_{i,m}p_{i+1,n}\left(i\leq m\leq 20,1\leq n\leq 20\right)$$

From the equation, we can get a bi-gram matrix with 400 elements. The 400 elements in matrix *B* are also the feature vectors of the protein fold recognitions that we need. Let *F* be the bi-gram feature vector of the protein fold recognition, which is as follows:

(4) $$F={\left\{b_{1,1},b_{1,2},\ldots ,b_{1,20},b_{2,1},\ldots ,b_{2,20},\ldots ,b_{20,1},\ldots ,b_{20,20}\right\}}^{T}$$

where the symbol *T* means the transpose of the feature vector [33].

It is intuitively plausible that the bi-gram feature *F* contains much more serviceable information of protein fold recognitions than extracting bi-gram features directly from the primary protein sequences or the consensus protein sequences. Generally speaking, from a biological point of view, proteins with the same physical folds have highly conserved amino acid subsequences [34]. In these conserved areas, the subsequences of amino acids are represented by the bi-gram probability features [35]. Consequently, if a certain subsequence of amino acids is known to be conservative in a protein fold, there is a group of bi-gram features that represent each protein in the fold from that conserved area. This protein character can help us to distinguish those protein folds from different amino acid subsequences [36].

### 4.4. Representation of ncRNA Sequences Using K-mers Sparse Matrix and SVD 

As for ncRNA, we selected the deformation of two-dimensional matrices k-mers sparse matrices to store the features of the ncRNA sequences [24]. Using two-dimensional matrices to represent ncRNA sequences instead of one-dimensional vectors, much more useful and significant information is stored in the original sequences, such as location information, can be saved. Thus, the ncRNA features that were obtained by the two-dimensional matrix ought to have higher accuracy and better performance in predicting the interactions between ncRNAs and proteins [24].

A ncRNA sequence is processed into a $4^{k}\times \left(L-k+1\right)$ k-mers sparse matrix *M*. When $m_{j}m_{j+1}m_{j+2}m_{j+3}$ is just equal to the $i_{th}$ k-mers among $4^{k}$ different k-mer, set the element $a_{i,j}=1$. Subsequently, the matrix *M* can be defined, as follows:

(5) $$M={\left(a_{ij}\right)}_{4^{k}}\times \left(L-k+1\right)$$

(6) $$a_{ij}=\{\begin{array}{l} 1,if\;m_{j}m_{j+1}m_{j+2}m_{j+3}=k-mer\left(i\right)\\ 0,\;else \end{array}$$

After obtaining the corresponding two-dimensional matrix from the original sequence of ncRNA, we transformed this matrix with large amounts of data by using SVD [37]. There are two primary functions of applying SVD on the matrices. The first is Low Rank Approximation, the essence of which is an approximate method that transfers the original complex matrix into a suitable corresponding low rank matrix by way of low dimensional structures in the high dimensional space. Through this efficient approximate technique, more valuable properties and information from the original complex matrices can be conserved in new matrices [24]. In addition, the redundant information and noise can be effectively reduced. Besides, storage space, as well as computational complexity, can be reduced further more. The second is dimensionality reduction. As the dimension increases, the limited sample space becomes sparse, contributing to a phenomenon in which the model lacks generalization capability for the new data in spite of it performing well on the training set data. Feature dimension reduction is proposed to reduce the dimension and eliminate over-fitting phenomenon.

### 4.5. Stacked Auto-Encoder and Fine Tuning

The function of deep learning is learning various expressions of raw data layer by layer. Each layer extracts more abstract and suitable complex features that are based on the expression features of the previous layer to do some classification tasks. Actually, SAE is an unsupervised feature learning approach that does the same thing as a member of large proportions of deep learning. In simple terms, the structure of SAE is to stack multiple layer auto-encoders layer by layer [38]. Both the sparse auto-encoders and the de-noising auto-encoders are kinds of mono-layer auto-encoders to learn a characteristic change of

(7) h=f(Wx+b)

through a three layer network x→h→x. The input *x* can be interpreted as a *d*-dimension dataset and *f* can be interpreted as a non-linear function in the expression, which is an element-wise sigmoid function $f\left(x\right)=\frac{1}{1+e^{-x}}$ here.

In this study, the types of layer that we used are the dropout layer and fully connected layer [39]. In the dropout layer, some neurons unit activations are set to be zero randomly to avoid model training over-fitting. After using SAE system as an unsupervised learning, a fine tuning operation is used to tune each parameter of each layer based on back-propagation for a better performance. The SAE system can be improved a lot through the use of fine tuning. In the fine tuning operation, a softmax layer as the last layer with a sigmoid function is added to output from merged sub-networks of ncRNA and protein as the expression.

Therefore, a SAE model can be formulated as the following optimization formula:

(8) $$\min\left[\overset{a}{\underset{i=1}{\sum }}{\left(h_{W,b}\left(x^{\left(i\right)}\right)-y^{\left(i\right)}\right)}^{2}+\alpha \left({\left\|W\right\|}^{2}\right)+\beta \overset{b}{\underset{j=1}{\sum }}KL\left(p\|{\hat{p}}_{j}\right)\right]$$

where ${\hat{p}}_{j}$ is the mean activation probability in the $j_{th}$ hidden unit, which calculated from ${\hat{p}}_{j}=\frac{1}{a}\sum_{i=1}^{a}h_{j}$ and the element *p* represents the desired probability of being activated.

In (8), the first item represents the reconstruction cost, the second item refers to a regularization on weight to avoid over-fitting, and the last item makes a sparsity mapping from input layers to hidden layers, in which the Kullback–Leibler (KL) divergence is employed to measure the similarity between the desired and actual values shown as follows [40]:

(9) $$KL\left(p\|{\hat{p}}_{j}\right)=p\log\frac{p}{{\hat{p}}_{j}}+\left(1-p\right)\log\frac{1-p}{1-{\hat{p}}_{j}}$$

In the process of training datasets, the dropout probability is set to be 0.5, which is the most suitable probability of dropout training system [41]. The whole SAE system is realized by the use of keras library, while its parameters *batch_size* and *nb_epoch* is both set to be 100. The keras library is described in detail in the website http: //github. com/fchollet/keras. 

### 4.6. Stacked Ensembling

For most classifiers, there are different classification performances to adapt to different kinds of classification problems. To acquire the approximate optimal objective functions, it is necessary to use multiple classifiers to integrate learning. Accordingly, it is crucial to find out the solution of ensembling mechanism implementing to integrate the individual output. In the previous studies, most of the solutions are the average individual model results strategy and the majority voting strategy [42,43]. While, in stacked ensembling, the output of the level 0 classifier will be used as training data for the level 1 classifier as the combining strategy of multiple layers of neural networks intuition, where level 0 is the first layer in stacked ensembling and level 1 is the successive layer after level 0. The function of the level 1 classifier is trying to combine the results of all single classifiers [44]. In this study, the outputs of all level 0 classifiers are interpreted as predicted probability scores, while the successive level 1 classifiers are logistic regression classifiers. As a result, the stacked ensembling is equal to the average individual model result strategy when the score weights of logistic regressions of all individual level 0 classifiers are the same, while it is equal to the majority voting strategy when there is only one score weight that becomes non-zero [17].

(10) $$P_{w}\left(y=\pm 1|s\right)=\frac{1}{1+e^{-yw^{T}s}}$$

where *s* represents predicted probability scores of all level 0 classifiers vector outputs and *w* is the weight vector of corresponding classifiers. The logistic regression is obtained from Scikit-learn [45].

### 4.7. Prediction Methods and Evaluation Criteria

In the machine learning field of all classifiers, there are four representative classifiers that are supervised and efficient, including the SVM [6], neural network [46,47], Naïve Bayesian [48], and RF [42]. Those four classifiers are managed and compared with each other, aiming to select the appropriate one with the best accuracy and performance for predicting ncRNA and protein interactions. In BGFE, to classify and predict, a RF classifier was used.

RF refers to a classifier that uses multiple decision trees to train and predict datasets [49]. In machine learning, the RF classifier is a multiple decision tree classifier with its output composed of plural individual tree categories depending on the output category [50]. In order to construct RFs, the datasets need to be selected from the random characters randomly, which guarantees the difference of the decision trees in the RF classifier. Furthermore, the diversity of the system can be enhanced and the classification performance can be improved as well. In order to actualize this target, a five-fold cross validation technique is employed for evaluating algorithm performance and accuracy about each group of dataset [51]. In the python environment, we can use the Scikit-learn package to help us complete the task of constructing RFs [45].

In this study, several widely used computational criteria was employed to evaluate the prediction performance, as follows [52]:

(11) $$Accuracy=\frac{TP+TN}{TP+TN+FP+FN}$$

(12) $$Sensitivity=\frac{TP}{TP+FN}$$

(13) $$Specificity=\frac{TN}{TN+FP}$$

(14) $$Precision=\frac{TP}{TP+FP}$$

(15) $$MCC=\frac{TP\times TN-FP\times FN}{\sqrt{\left(TP+FP\right)\left(TP+FN\right)\left(TN+FP\right)\left(TN+FN\right)}}$$

where *TP*, *FP*, *TN,* and *FN* are, respectively, interpreted as the numbers of true positive, false positive, true negative, and false negative. The ROC curve is understood as the threshold between *specificity* and *sensitivity*. Meanwhile, the AUC is regarded as the area of the graph under the ROC curve. As a result, the probability of falsely predicting the interaction between ncRNA and protein pairs will be no more than one percentage.

## 5. Conclusions

In this study, a sequence-based method named BGFE using deep learning model SAE network combined with the Random Forest classifier was proposed. We used k-mers sparse matrices to represent ncRNA sequences, and then extracted feature vectors from these matrices by SVD. For protein sequences, PSSM was used to obtain evolutionary information from each sequence; moreover, the bi-gram algorithm was further used to get feature vectors from PSSM. We selected three public datasets to evaluate the performance of our model. In the experiments, our method BGFE achieved a great performance in protein-ncRNA interactions in predicting tasks when the experiment results and capability were evaluated. We also made a comparison between BGFE and other current methods, while our method obtained a better performance than other methods in predicting interactions between ncRNA and proteins. This study can predict the potential non-coding RNA-protein interacted pairs accurately and it provides some useful guidance for the future biological research.

## Figures and Tables

**Table 1:** Prediction Performance on Dataset RPI488, RPI1807, and RPI2241.

Dataset	Accuracy	Sensitivity	Specificity	Precision	MCC
RPI488	0.8868	0.9268	0.8354	0.9328	0.7743
RPI1807	0.9600	0.9344	0.9989	0.9117	0.9217
RPI2241	0.9130	0.8772	0.9660	0.8590	0.8335

**Table 2:** Specific Performance of Four Methods on Dataset RPI488.

RPI488	Accuracy	Sensitivity	Specificity	Precision	MCC
BGFE	**0.8868**	**0.9268**	**0.8354**	0.9328	**0.7743**
Raw feature	0.8168	0.8083	0.8192	0.8104	0.6299
Stacked auto-encoder	0.8806	0.9243	0.8255	**0.9351**	0.7638
Stacked auto-encoder without fine tuning	0.8600	0.8848	0.8271	0.8850	0.7187

The boldface indicates this measure performance is the best among the compared methods for individual dataset.

**Table 3:** Specific Performance of Four Methods on Dataset RPI1807.

RPI1807	Accuracy	Sensitivity	Specificity	Precision	MCC
BGFE	0.9600	0.9344	0.9989	0.9117	0.9217
Raw feature	0.9349	0.9508	0.9308	0.9400	0.8688
Stacked auto-encoder	0.9396	0.9029	**0.9994**	0.8651	0.8830
Stacked auto-encoder without fine tuning	**0.9645**	**0.9672**	0.9688	**0.9590**	**0.9281**

The boldface indicates this measure performance is the best among the compared methods for individual dataset.

**Table 4:** Specific Performance of Four Methods on Dataset RPI2241.

RPI2241	Accuracy	Sensitivity	Specificity	Precision	MCC
BGFE	**0.9130**	0.8772	**0.9660**	0.8590	**0.8335**
Raw feature	0.6438	0.6525	0.6313	0.6565	0.2881
Stacked auto-encoder	0.9041	**0.8895**	0.9329	**0.8747**	0.8156
Stacked auto-encoder without fine tuning	0.6438	0.6517	0.6327	0.6551	0.2879

The boldface indicates this measure performance is the best among the compared methods for individual dataset.

**Table 5:** The Performance Comparison between BGFE and Other Methods on Dataset RPI1807 and RPI2241.

RPI1807	Accuracy	Sensitivity	Precision
BGFE	0.9600	0.9344	0.9117
RPI-Pred	0.9300	0.9400	0.9400
**RPI2241**	**Accuracy**	**Sensitivity**	**Precision**
BGFE	0.9130	0.8772	0.8590
RPI-Pred	0.8400	0.7800	0.8800
Usha K Muppirala	0.8960	0.9000	0.8900
Ying Wang	0.7400	0.9160	0.6990

**Table 6:** Details of the ncRNA-Protein Interaction Datasets.

Dataset	Interaction Pairs	Number of Proteins	Number of RNAs
RPI488 ^1^	243	25	247
RPI1807 ^1^	1807	1807	1078
RPI2241 ^1^	2241	2043	332

^1^ RPI488 is lncRNA-protein interactions based on structure complexes, RPI2241 and RPI1807 are RNA-protein interactions.
